# Advanced Imaging of the Vestibular Endolymphatic Space in Ménière's Disease

**DOI:** 10.3389/fsurg.2021.700271

**Published:** 2021-08-23

**Authors:** Diego Zanetti, Giorgio Conte, Elisa Scola, Silvia Casale, Giorgio Lilli, Federica Di Berardino

**Affiliations:** ^1^Audiology Unit, Department of Specialistic Surgical Sciences, Fondazione IRCCS Ca' Granda Ospedale Maggiore Policlinico, University of Milan, Milan, Italy; ^2^Audiology Unit, Department of Clinical Sciences and Community Health, Fondazione IRCCS Ca' Granda Ospedale Maggiore Policlinico, University of Milan, Milan, Italy; ^3^Neuroradiology Unit, Fondazione IRCCS Ca' Granda Ospedale Maggiore Policlinico, Milan, Italy

**Keywords:** magnetic resonance imaging, hydrops, membranous labyrinth, ear, vestibule

## Abstract

The diagnosis of “definite” Méniére's disease (MD) relies upon its clinical manifestations. MD has been related with Endolymphatic Hydrops (EH), an enlargement of the endolymphatic spaces (ES) (cochlear duct, posterior labyrinth, or both). Recent advances in Magnetic Resonance (MR) imaging justify its increasing role in the diagnostic workup: EH can be consistently recognized in living human subjects by means of 3-dimensional Fluid-Attenuated Inversion-Recovery sequences (3D-FLAIR) acquired 4 h post-injection of intra-venous (i.v.) Gadolinium-based contrast medium, or 24 h after an intratympanic (i.t.) injection. Different criteria to assess EH include: the comparison of the area of the vestibular ES with the whole vestibule on an axial section; the saccule-to-utricle ratio (“SURI”); and the bulging of the vestibular organs toward the inferior 1/3 of the vestibule, in contact with the stapedial platina (“VESCO”). An absolute link between MD and EH has been questioned, since not all patients with hydrops manifest MD symptoms. In this literature review, we report the technical refinements of the imaging methods proposed with either i.t. or i.v. delivery routes, and we browse the outcomes of MR imaging of the ES in both MD and non-MD patients. Finally, we summarize the following imaging findings observed by different researchers: blood-labyrinthine-barrier (BLB) breakdown, the extent and grading of EH, its correlation with clinical symptoms, otoneurological tests, and stage and progression of the disease.

## Introduction

Ménière's disease (MD) is thought to be an unbalance of inner ear fluids, manifesting as fluctuating sensorineural hearing loss (SNHL), recurrent vertigo spells, tinnitus, and ear fullness (1). It affects between 200 and 500 persons every 100,000 inhabitants in Western countries (2). Endolymphatic Hydrops (EH), a swelling of the endolymphatic space (ES) that occupies part of the perilymphatic space (PS) constitutes the pathological landmark of the disease (3). It encompasses the cochlear duct and the saccule, sometimes extending to the semicircular canals (SCC) and utricle.

In 1995, the American Academy of Otolaryngology—Head and Neck Surgery (AAO-HNS) re-established the guidelines for the assessment of MD (4). In 2015 (5), a new consensus was reached on 2 main forms of MD “definite” and “probable” MD. Diagnosis of **definite** MD is obtained upon two or more vertigo spells limited to a period between 20 min and 12 h, associated with unilateral low-frequencies SNHL, tinnitus and intermittent ear fullness. **Probable** MD is defined by more than 2 vertigo spells lasting between 20 min and 1 day and ipsilateral intermittent ear fullness. To evaluate the presence of EH, electrocochleography (ECochG) is able to identify, through an acoustic stimulation, the increase of the endolymphatic pressure with bulging of the Reissner's membrane in case of EH. Other electrophysiological tests, such as the vestibular evoked myogenic potentials (VEMPs) and video head impulse test (vHIT) might be useful, but their role is still controversial.

Recent reports confirm that the otoneurological responses correlate well with the magnetic resonance imaging (MRI) showing that the EH starts from the saccule and then progresses to the SCC (6).

In the past, imaging studies were still obtained to exclude retrocochlear disorders, such as vestibular nerve schwannomas. New developments of MRI techniques (7) have enabled visualization of EH in humans by means of 3 Tesla (3T) scanners and Gadolinium (Gd) administered either intravenously (i.v.) (8) or intratympanically (i.t.) (9, 10). However, the diagnostic accuracy of EH by MRI is disputed, mainly owing to the disparities in the inclusion and diagnostic criteria.

The clinical consequences of a correct assessment of EH would represent a significant leap forward, by providing a more reliable tool to differentiate between MD and non-MD diseases of the inner ear (11, 12) to follow-up the untreated clinical evolution of the disease (13) or to test different pharmacological or surgical protocols (14, 15). The purpose of this review of the literature is to understand the reliability of MRI in detecting EH in the population of suspected MD patients with insights of the contemporary technological advances and to compare the imaging findings with the natural history of the disease and the outcome of treatments. Furthermore, we discuss the newest MR protocols to assess EH and the commonest MRI findings in different vestibular disorders.

### Literature Search Strategy

According to the PRISMA guidelines (16), the scientific literature of the last decade was browsed with the aim of identifying studies describing the MR findings in “definite” MD patients (see the PRISMA flowchart in the additional digital content).

The following search queries were used in Medline, Cochrane database, Scopus and EMBASE (from 2010 to Present): “*Ménière*” *OR* “*endolymphatic hydrops*” AND “*magnetic resonance*” OR “*MR*.” The search was restricted to articles that provided at least an abstract in English. References of the selected publications were also examined to extract any further relevant article. The retrieved articles were considered eligible if they provided results of MR in patients with “definite” MD (4) or to the 2015 Consensus Statement (5). Two of the co-Authors (FDB and GL) separately reviewed all articles and excluded those with unclear clinical diagnostic criteria. Other exclusion criteria were studies on animals, case reports, personal (expert) opinions, metanalysis, different diagnostic criteria, studies where diagnosis of “definite” MD was lacking; studies not assessing hydrops by accepted MRI techniques; and studies not reporting the status of the contralateral (unaffected) ear.

The following information were outsourced from the selected studies: first author, year of publication, total number of subjects enrolled, MRI techniques and peculiar MRI findings related with the presence of hydrops in the affected vs. unaffected ears.

### Literature Search Results

The initial search yielded a total of 219 articles: 97 articles in PubMed, 91 in EMBASE, 27 in Scopus and none in the Cochrane Library. After excluding duplicates and those not respecting the inclusion criteria, 77 articles were left; among these, 47 were included by relevance. These 47 papers were carefully analyzed for the purposes of this study. Six of them were review studies and were only considered for the general overview of the topic.

The main features of each of the 41 remaining articles are reported in Table 1: 8 out of 41 studies performed an i.t. administration of Gd, 27 only an i.v. delivery and 3 studies performed both simultaneously; 3 studies dealt with post-acquisition processing of images.

**Table 1:** Summary of literature review (last decade) on MRI in Meniere's disease (MD).

**References**	**year**	**# of pts**	**MRI method**	**MR findings**	**Notes**
Fiorino et al. (10)	2011	32	3T 3D-FLAIR after i.t. Gd	Degree of EH in cochlea and the semicircular canals directly proportional to MD duration	
Gürkov et al. (17)	2012	41	3D IR-TSE	Correlation of EH (Likert scale) with duration of MD, degree of SNHL, saccular dysfunction	ECochG, VEMPs, VNG
Claes et al. (14)	2012	12	3T 3D-FLAIR after i.t. Gd	Correlation between TT-ECoG and EH grading on MRI	
Naganawa et al. (18)	2012	24	3T 3D-FLAIR after i.v.Gd → HYDROPS2	Subtraction of MR cisternography from PPI facilitated recognition of the ES	Scan time 40% < than HYDROPS
Sano et al. (19)	2012	10	3T 3D-FLAIR after i.v.Gd 4 h delayed acquisition	Increased Gd enhancement in symptomatic ears.	Better resolution after 4 h
Naganawa et al. (20)	2013	10	HYDROPS-Mi2 after i.v. Gd	Multiplied MRC onto HYDROPS → better visualization of ES and PS on a single volumetric image in EH+ pts	CNR ratio increase 200 fold
Seo et al. (21)	2013	26	3T 3D-FLAIR 24 h after IT.Gd	EH in the cochlea (81%) and saccule (69%). Correlation with auditory and vestibular testing	Audiometry, VEMPs, ECochG
Naganawa et al. (22)	2014	10	i.t. Gd Real-IR + i.v. 3D FLAIR	Superiority of combined administration vs. i.v. Gd alone	
Barath et al. (23)	2014	53	i.v. Gd 3T 4 h 3D-real IR	EH in 90% on the clinically affected and in 22% on the clinically silent side	
Liu et al. (24)	2015	30	3T 3D-FLAIR after IT Gd	EH also in 23.3% of asymptomatic ears	
Sepahdari et al. (25)	2015	41	i.v. Gd 3D-FLAIR + MIP	MIP superior to 2D images for EH assessment	
Suga et al. (13)	2015	12	3T 3d-FLAIR 24 h after i.t. Gd + 4 h after i.v. Gd	EH reduction at 1 yr correlates with symptoms recovery	
Hornibrook et al. (26)	2015	57	3T 3d-real IR 24 h after i.t. Gd	High correlation between ECochG (tone bursts) and EH at MRI	vs. 45 other pathologies
Bykowski et al. (27)	2015	6	3T 2d-FIESTA + T1SE 24 h after i.t. Gd	Using a 3-inch surface coil preserves high resolution within a clinically acceptable acquisition time	
Attyé et al. (28)	2015	132	3T 3d-FLAIR 4 h after i.v. Gd	Similar pathophysiological mechanism in RPV and MD	EH in RPV/MD
Wu et al. (29)	2016	54	3T 3D-FLAIR vs. 3D-real IR after i.t. Gd	Low-tone hearing thresholds correlates with severity of EH in the cochlea.	EH progressed over time.
Pakdaman et al. (30)	2016	32	3T 3d-FLAIR 4 h after i.v. Gd + T2 SPACE	Increased BLB permeability in MD	vs. 11 pts with sudden SNHL
Keller et al. (31)	2017	85	3D FIESTA + 2D SPACE	Conventional MRI without Gd allows detection of EH	
Choi et al. (32)	2017	46	3T 3D-FLAIR MRC after i.v.Gd and HYDROPS-Mi2	Degree of EH in MD vs. VN patients	Correlation with caloric tests
Attyé et al. (11)	2017	30	3T 3d-FLAIR 4 h after i.v. Gd	SURI: most specific criterion for imaging of EH	vs. 30 healthy ear
Okumura et al. (33)	2017	21	3T CISS + 2d-FLAIR 4 h after i.v. Gd	70% EH + low correlation with VEMPS and VNG	
Yoshida et al. (34)	2017	42	3T 3d-FLAIR 4 h after i.v. Gd	Prevalence of vestibular EH significantly higher in MD	vs. healthy ears
Conte et al. (12)	2018	22	3T 3D-FLAIR 4 h after i.v. Gd	New criterion of EH assessment: VESCO	vs. normal ears.
Wesseler et al. (35)	2018	31	3T 3D-FLAIR-SPAIR 24 h after i.t. Gd	MRI more accurate than caloric test, vHIT, and cVEMP.	MR scan for hydrops after 24 h
Bier et al. (36)	2018	10	MRC+VISTA-IR+ HYDROPS 4 h after i.v. Gd	EH gradient along the cochlea	
Quatre et al. (37)	2019	41	3T 3D-FLAIR 4 h after i.v. Gd	Comparison with audio-vestibular tests: ECochG and DPOAEs correlated with EH	ECochG, shift-DPOAE, cVEMPs
Pérez-Fernández et al. (38)	2019	22	3T 3D-FLAIR 4 h after i.v. Gd	Degree of vEH correlates with vestibular deficit	vHIT, VEMPs, audiometry
Bernaerts et al. (39)	2019	148	3T 3D-FLAIR 4 h after i.v. Gd	4 degrees of EH. Combined with perilymphatic enhancement: Sensitivity 79.5% Specificity 93.6%	
Ito et al. (15)	2019	20	3T 3D-FLAIR + MRC 4 h after i.v. Gd	Reduction of EH 2 ys after endolymphatic sac drainage and local steroids	pre- and post-surgery MR
Eliezer et al. (40)	2019	30	3T 3D-FLAIR + SSFP 4 h after i.v. Gd	In acute vestibular deficits	vs. healthy ears
Guo et al. (41)	2019	56	3T T2-SPACE and Real-IR 4 h after i.v. Gd	Degree of EH correlates with audiometry and VEMPs	oVEMP and PTA correlates with cochlear EH
Ohashi et al. (42)	2020	15	HYDROPS-Mi2 + 3D-real IR images	Measurement of endolymphatic volume	Comparison of 2 techniques
Gerb et al. (43)	2020	105	3T 3D-FLAIR + CISS 4 h after i.t. Gd	VOLT: new algorithm for automatic segmentation of MR images	All pts with acute vertigo (incl. MD)
Nahmani et al. (44)	2020	16	3T 3D-FLAIR 4 h after i.v. Gd Variable Flip Angle-	Reliable in evaluating BLB breakdown and ES	
Cho et al. (45)	2020	226	INHEARIT on archived datasets	Feasibility of automated EH ratio measurements	Automatic segmentation + calculation of EH
van Steekelenburg (46)	2020	220	3T 3D-FLAIR + SPACE 4 h after i.v. Gd	EH in 91.9% of MD vs. 7% in other vertigo.	Combination of PPI and EH in MD
Pai et al. (47)	2020	31	3T 3D-FLAIR 4 h after i.v. Gd	Correlation of EH finding at MR with diseased ear	
Fukushima et al. (48)	2020	55	3T 3D-FLAIR and HYDROPS-Mi2 4 h after i.v. Gd	Progression of vestibular EH over 2–3 years	MR repeated annually
Kahn et al. (49)	2020	31	3T 3D-FLAIR 4 h after i.v. Gd	EH: Cochlear 88%, saccular 91%, utricular 50%, ampullar 8.5%. No correlation with VEMPs/vHIT. Severity of EH correlated with SNHL	vs. 26 healthy ears
Gürkov et al. (50)	2021	30	1.5T 3D-FLAIR Hydrops 24 h after i.t. Gd	Reliability of 1.5T fast identification and grading of EH	
He et al. (51)	2021	50	3T 3D-FLAIR 4 h after i.v. Gd	3D-FLAIR MRI + PT- ECochG more sensitive than ECochG alone for EH	PT- ECochG
Zhang et al. (52)	2021	24	3T 3D-FLAIR 4 h after i.v. Gd	Degree of EH correlates with hearing threshold	Extratympanic
Sluydts et al. (53)	2021	78	3T 3D-FLAIR 4 h after i.v. Gd	Only severe cochlear and vestibular EH are associated with cochleovestibular dysfunction	Audiometry, caloric tests, VEMPs, vHIT

*BLB, blood-labyrinth barrier; CISS, continuous Interference Steady-State; CNR, contrast-to-noise ratio; ECochG or TT-ECochG, trans-tympanic electro-cocleography; EH, endolymphatic hydrops; ES, endolymphatic space; FLAIR, Fluid Attenuated Inversion Recovery; hT2w-VISTA-IR, heavily T2-weighted volume isotropic turbo spin echo acquisition inversion recovery; HYDROPS, Hybrid of the reversed image of the positive endolymph signal and native image of positive perilymph signal; HYDROPS-Mi2, multiplication of HYDROPS with MRC; INEARHIT, Inner Ear Hydrops Estimation via Artificial Intelligence; MIIRMR, medium inversion time inversion recovery imaging with magnitude reconstruction; MIP, maximum intensity projections; IR-TSE, Inversion Recovery Turbo Spin-Echo; MRC, magnetic resonance cisternography; PEI, positive endolymph image; PPI, positive perilymph image; PS, perilympahtic space; PT- ECochG, peri-tympanic electro-cocleography; RPV, Recurrent peripheral vestibulopathy; SNHL, sensorineural hearing loss; SPACE, sampling perfection with application-optimized contrasts by using different flip angle evolutions; SPAIR, Spectral Attenuated Inversion Recovery; SSFP, steady-state free precession sequences; SURI, Inversion of the saccule to utricle area ratio; 3D-REAL-IR, 3D-inversion-recovery turbo spin-echo with real reconstruction; VEMPs, vestibular-evoked myogenic potentials (oVEMPs: ocular, cVEMPS: cervical); VESCO, vestibular endolymphatic space contacting the oval window; VHIT, video Head Impulse Test; VNG, video-nystagmography; VN, vestibular neuritis*.

The MR images were acquired 4 h after the i.v administration in 26 out of 27 studies, and 24 h after the i.t. injection in 8 out of 11 trials (8 i.t. only and 3 i.t. + i.v).

A 3T machine was used in 40 out of 41 studies. The most common MRI technique to identify EH was the 3D FLAIR (*n* = 38/41 original studies).

The selected method to assess the degree of EH was the VES/vestibule ratio > 30% in 28 studies, > 50% in 3 studies, the SURI in 4 studies, the VESCO only in 1 study.

The sensitivity, specificity, PPV and NPV of the techniques in correctly identifying EH were reported in 37 out of 41 studies.

Side effects of Gd administration were reported in 3 of 1,578 patients (sum of all articles), all of them with the i.t. routes.

## Discussion

The published papers on MRI of the inner ear have almost doubled during the last 10 years, confirming the interest in the subject and the potentiality of the innovative methods. However, the role of MRI in differentiating between different inner ear diseases remains to be established. Based on general opinion in the field, we considered reasonable to report on the volumetric detection of EH rather than generic imaging of MD.

The MRI identification of the fluid compartments of the inner ear can be achieved by either intratympanic or intravenous administration of Gd (8).

### The Intra-Tympanic Gadolinium Injection

It has been the first method proposed in the literature to study the inner ear spaces (9, 54). The i.t.-Gd delivery method utilizes an i.t. injection of 0.4–0.7 ml of an 8-fold dilution of the Gd solution into the tympanic cavity. The contrast medium diffuses into the perilymph through the round window membrane (RWM), but not in the endolymph, producing the so-called perilymph positive image (PPI). Considering the diffusion dynamics, the MRI scans are obtained 1 day after the i.t. injection. The method of choice is based on T2-weighted scans with the 3D Fluid-Attenuated-Inversion-Recovery (3D-FLAIR) algorithm, which lowers the endolymph signal in respect to the adjacent perilymph. Variations to the flip angle can be applied. If the inversion time of the 3D-FLAIR is shortened, the perilymph signal is suppressed and that of the endolymph is enhanced, obtaining a positive endolymphatic image (PEI). Another technique, namely 3D-inversion-recovery turbo spin-echo (TSE) with real reconstruction (3D-real IR) (55) creates a sharp contrast between the inner ear fluids (the positive perilymph vs. the negative endolymph) and the neighboring bone that results as null.

Given the known entry routes and kinetics of drugs from the middle to the inner ear, the most suitable and reliable method to investigate the vestibular EH (vEH) and the cochlear EH (cEH) is that described by Nakashima et al. (9). In contrast, Carfrae et al. (54) and Shi et al. (55) denied an additional value of Gd delivered directly to the middle ear during surgery: vEH and cEH were detected only in 25 and 16% of patients, respectively, possibly owing to the dilution of the contrast medium. In many countries, the i.t. delivery of Gd is still off-label; moreover, some patients are reluctant to undergo bilateral injections of their tympanic membranes. Thus, the puncture is limited to the affected ear, leaving the contralateral uninvestigated.

Wu et al. (29) administered a bilateral i.t. injection of Gd: the presence of vEH was detected in two thirds of the ears with clinical symptoms of MD, while cEH in 8%. A few studies tested the simultaneous delivery of Gd through the i.t. and the i.v. routes: Iida et al. (56) demonstrated the presence of both vEH and cEH in 67% of the contralateral asymptomatic ears. Naganawa et al. (22) used 3D-real IR images for the i.t.-Gd side and the so-called HYDROPS sequences (“*Hybrid of the reversed image of the positive endolymph signal and native image of positive perilymph signal*”) for i.v perfusion. Only HYDROPS images were able to demonstrate vEH in 89% and cEH in 67% of all symptomatic ears, respectively. The i.t. administration is able to show the presence of EH in other audiological disorders (26, 57), even if at a very low rate.

To improve the acquisition and analysis of the images, Bykowski et al. (27) applied 8-channel surface coils to acquire 3D-FLAIR images after an i.t. injections in six patients with definite MD. By varying the inversion times, they were able to judge the fluid-suppression ability of each sequence, in all the six patients tested.

### The Intra-Venous Gadolinium Perfusion

Although the i.t.- Gd technique has the great benefit of enhancing the visualization of the perilymph (35), the i.v.-Gd delivery route has several advantages: lesser invasiveness (although major complications can still occur); reduced operating times (4 h for a comprehensive MRI study); bilateral examination in a single test session. It consists in the intravenous perfusion of a fixed (per weight) dose of Gd (between 0.1 and 0.2 ml/Kg) that rather quickly diffuses in the perilymph without spreading to the endolymph, depending on the permeability of the blood-labyrinth barrier (BLB) (58), thus creating a PPI. The 2 most popular scanning sequences, 3D-real IR and 3D-FLAIR are substantially equivalent, although the latter is more sensitive to less concentrated dilutions of Gd (12).

Noteworthy, a correct visualization of the inner ear compartments relies upon the inversion time. After the normal MR standards are established in healthy controls, MR images can be immediately analyzed after acquisition or they can be scrutinized after processing. Post-processing includes subtraction of the PEI (a 3D FLAIR sequence) from the PPI. HYDROPS and HYDROPS2 (22) are then reconstructed and can be juxtaposed to the corresponding MR cisternography images to obtain the HYDROPS-Mi2 and HYDROPS2-Mi2 images, respectively, which show increased contrast compared to background noise (42). The combination of “*maximum intensity projection*” (MIP) and 3D-FLAIR further adds robustness to the assessment of EH.

The main concern about either technique of MRI of EH is the correct identification of the vestibular organs, which requires a thorough knowledge of the radiological anatomy. During the last decade, a number of different methods to detect hydrops have been proposed, raising also a vivid debate among research groups.

Nakashima et al. (59) initially described the vestibular endolymphatic space (VES) by calculating the ratio between the endolymphatic organs and the whole vestibule in an axial projection. They defined the vEH as “*absent*” when the ratio was <33%, “*mild*” when 34-50% and “*significant*” if >50%. In addition, they evaluated the cochlear ES (cES) by measuring the displacement of the Reissner's membrane. They defined the cEH as “*mild*” if the displacement did not exceed the scala vestibuli (SV), or “*significant*” when the cES exceeded the SV.

In clinical studies, the first problem encountered with the VES/vestibule ratio was that mild vEH was not only reported in the greatest majority of symptomatic ears of MD patients, but also in more than half of the asymptomatic contralateral ears (19, 34, 58, 60). Moreover, EH has been detected also in healthy individuals (11, 61, 62), and this challenges its correlation with MD (63).

A few studies also raise the issue of specificity: mild vEH was identified even in symptomatic ears of patients with otological diseases other than MD (19), such as recurrent peripheral vestibulopathy and unilateral labyrinthine deficit (28, 40).

Nevertheless, the sensitivity of i.v. Gd-enhanced MR with 4 h delayed acquisitions is very high (>95%); thus, a significant VES/vestibule ratio might represent a potential indicator of MD even before a clear clinical diagnosis has been established. In fact, severe vEH was always excluded the ears of clinically silent MD patients (30) and mild vEH was in the very low range (below 20%) in healthy volunteers. Considering a VES/vestibule ratio > 50% (instead of 30%) may reduce its sensitivity but can represent a more reliable rule-in criteria for MD, allowing to differentiate MD from unaffected ears and from other auricular diseases.

Although Nakashima's criteria are universally accepted, other cut-off values for vEH have been suggested: Sepahdari et al. (25) used a VES/vestibule ratio of 45% (2 SD above the mean) in a group of patients with sudden SNHL and found vEH in 6/12 (50%), concluding that it was inconsistent for the assessment of MD in sudden SNHL. Another study (34) claimed that even a lower ratio of 41.9% yielded an absolute specificity of 100% in differentiating hydropic from normal ears, still retaining a high sensitivity (88.5%). They also studied the asymptomatic ears and ascertained cEH in 46% of MD patients and 33% of healthy subjects.

Other research groups graded the EH with a criterium based on the morphology of the saccule (11, 28, 40). Using a ratio between the area of the saccule and that of the utricle (SURI) >50%, they were able to differentiate the ears of 30 patients with MD from normal controls, with a sensitivity of 50% and specificity of 100. The reliability of post-contrast imaging in detecting EH was very high in Eliezer et al. study (64) by means of 3D-FLAIR and 4 h delayed acquisition, with much greater efficiency than the 3D FIESTA sequences.

**Figure 1:**
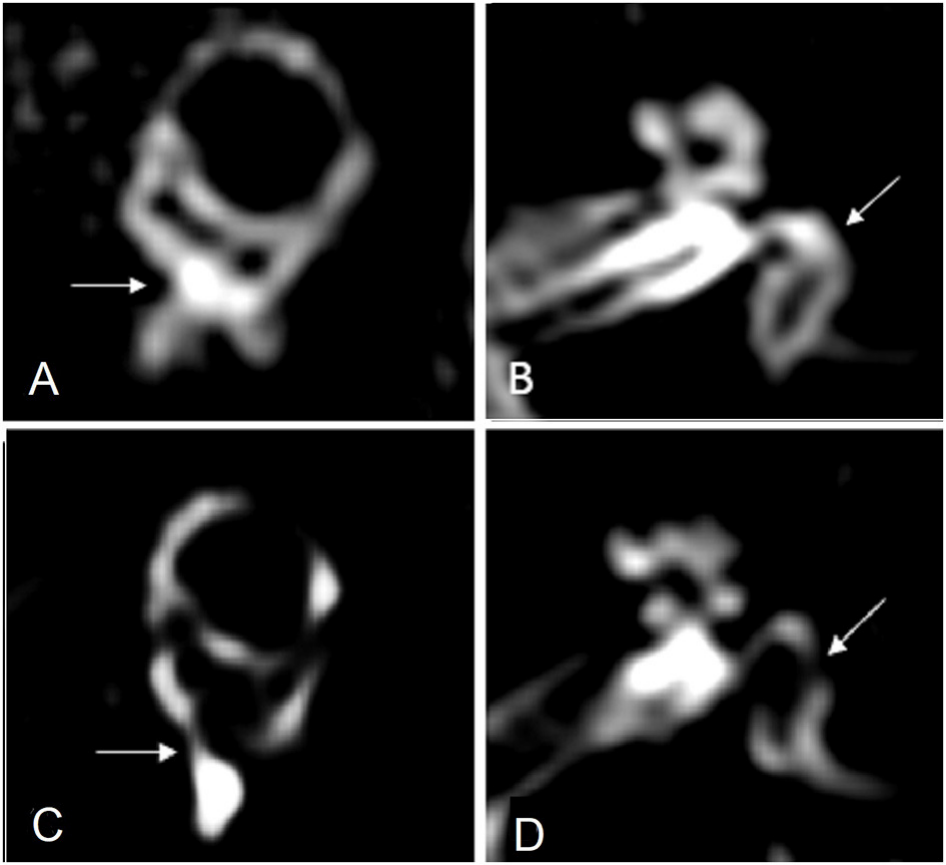
**(A)** MR imaging oblique sagittal reconstruction parallel to the superior semicircular canal of a healthy ear shows superiorly the vestibular endolymphatic space and inferiorly the perilymph filling the inferior third of the vestibule with preservation of the perilymph signal medial to the oval window (arrow). **(B)** MR imaging axial reconstruction parallel to the lateral semicircular canal at the inferior third of the vestibule in a healthy subject, showing the vestibule filled by the perilymph (arrow). **(C)** MR imaging oblique sagittal reconstruction parallel to the superior semicircular canal in a patient with Menière disease shows enlargement of the vestibular endolymphatic space bulging into the inferior third of the vestibule and contacting the oval window (arrow), with the consequent absence of the normal perilymph signal. **(D)** MR imaging axial reconstruction parallel to the lateral semicircular canal at the inferior third of the vestibule shows the vestibular endolymphatic space contacting the oval window, “VESCO” (arrow).

Conte et al. (12) compared the imaging outcomes in 49 subjects, half of which had suffered a sudden SNHL, and the remaining were afflicted by definite MD. Using the 4-h delayed 3D-FLAIR protocol, 2 independent examiners observed that in MD patients the saccule was swollen and protruded in the lower part of the vestibule, arriving in contact with the footplate of the stapes. They named this finding “VESCO” an acronym of “*vestibular endolymphatic space contacting the oval window*.” The VESCO showed an optimal specificity but a low sensitivity (81%) in differentiating MD ears from other inner ear diseases. Figure 1 details the regular membranous labyrinth anatomy in a 3T Gd-FLAIR axial scan in one of our healthy adult volunteers and depicts the VESCO in one of our symptomatic MD patients.

### Recent Advances

A number of technical innovations have been recently added to the MR armamentarium to improve the identification of EH, such as the Variable and Constant Flip Angle-Delayed 3D-FLAIR Sequences (44) or the i.t.-Gd “*medium inversion time inversion recovery imaging with magnitude reconstruction*” (MIIRMR) (65).

In order to eliminate the subjective (examiner's) bias, new automated images segmentation processing algorithms have been introduced (43, 66), also associated with deep-learning models based on Artificial Intelligence (45). By adding the quantification of perilymphatic enhancement to the grading of EH, van Steekelenburg et al. (46) recently reported to improve the positive predictive value of Gd-enhanced MRI from 0.92 to 0.97 in the confirmation of definite MD.

The majority of ongoing studies is currently aimed at targeting the correlation between the morphologic findings and the symptomatology (“the whole symptoms triad”) and, especially, with the results of the functional audio-vestibular testing and with the outcomes of treatment (37, 41, 48, 51, 52, 62, 63, 67). In general, the literature agrees that the presence of EH at MRI, independently from the cut-off values for definition of vEH, strongly correlates with the side of the disease in MD patients (47, 50), but it lacks specificity in differentiating MD from other inner ear disturbances, in the absence of clinical/instrumental confirmation (64). It seems that the MRI demonstration of EH is a necessary but not sufficient condition to assess a diagnosis of MD. Nevertheless, it is highly improbable that EH is only casually associated with MD, because the causative relationship is too stringent (2).

The present review has some limitations: the reported populations are rather heterogenous in diagnostic criteria and definition of MD; some studies include very small patients' samples, thus a metanalysis was not feasible. In contrast, the review offers an insight of the latest technological developments.

In conclusion, current MR imaging methods allow to clearly depict the ES vs. the PS, in both the cochlear and vestibular compartments with multiple dedicated protocols. MRI of the ES can be comfortably obtained by means of i.v. administration of Gd and late (4 h) acquisitions. Thus, the more invasive and off-label i.t. injection of Gd, is considered by most Authors a 2nd-line investigative tool.

Although the role of EH in MD has been questioned by recent research, and its presence may not always be considered pathological, current MR imaging yields a very high sensitivity in detecting it. As quantitative indexes alone are probably insufficient to establish an MRI diagnosis of MD, more accurate criteria based on the morphology of the endolymphatic organs are required. A 3 Tesla MR scanner is essential for the purpose of identifying the subtle morphological variations of the ES in MD and to correlate the findings with the clinical history and audio-vestibular testing. The newest techniques proposed in the last years appear to be promising tools and deserve to be further investigated, especially focusing on the features, prevalence and role of EH in different inner ear disorders and on the relevance of other findings (BLB breakdown, methemoglobin, and inflammatory deposits) in the ES.

## Author Contributions

GC defined the literature search strategy, evaluated the articles relevance, and revised the manuscript. ES scrutinized the retrieved articles for inclusion/exclusion in the systematic review. SC scrutinized the retrieved articles for inclusion/exclusion in the systematic review and provided the MR images with captions. GL prepared the draft of the manuscript. FDB revised the manuscript and checked references and table. DZ designed the study, revised, and edited the final version of the manuscript. All authors contributed to the article and approved the submitted version.

## Conflict of Interest

The authors declare that the research was conducted in the absence of any commercial or financial relationships that could be construed as a potential conflict of interest.

## Publisher's Note

All claims expressed in this article are solely those of the authors and do not necessarily represent those of their affiliated organizations, or those of the publisher, the editors and the reviewers. Any product that may be evaluated in this article, or claim that may be made by its manufacturer, is not guaranteed or endorsed by the publisher.
