# Supplementary figures and images for: Advanced Imaging of the Vestibular Endolymphatic Space in Ménière's Disease
Source: Front Surg. 2021 Aug 23;8:700271. doi: 10.3389/fsurg.2021.700271 (PMC8419327; doi:10.3389/fsurg.2021.700271)

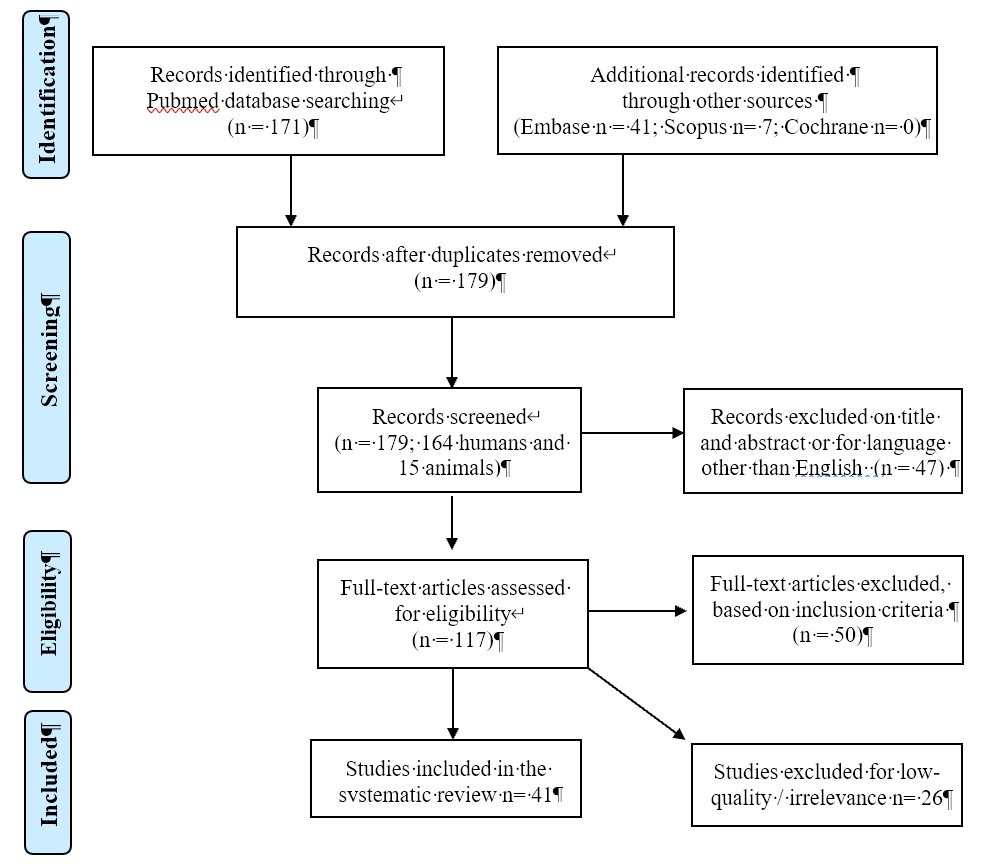

Supplement: Supplementary file 1 [file Image_1.JPEG]
